# The Geometric Increase in Meta-Analyses from China in the Genomic Era

**DOI:** 10.1371/journal.pone.0065602

**Published:** 2013-06-12

**Authors:** John P. A. Ioannidis, Christine Q. Chang, Tram Kim Lam, Sheri D. Schully, Muin J. Khoury

**Affiliations:** 1 Epidemiology and Genomics Research Program, Division of Cancer Control and Population Sciences, National Cancer Institute, National Institutes of Health, Bethesda, Maryland, United States of America; 2 Stanford Prevention Research Center, Department of Medicine and Department of Health Research and Policy, Stanford University School of Medicine, and Department of Statistics, Stanford University School of Humanities and Sciences, Stanford, California, United States of America; 3 Office of Public Health Genomics, Centers for Disease Control and Prevention, Atlanta, Georgia, United States of America; University of Hong Kong, Hong Kong

## Abstract

Meta-analyses are increasingly popular. It is unknown whether this popularity is driven by specific countries and specific meta-analyses types. PubMed was used to identify meta-analyses since 1995 (last update 9/1/2012) and catalogue their types and country of origin. We focused more on meta-analyses from China (the current top producer of meta-analyses) versus the USA (top producer until recently). The annual number of meta-analyses from China increased 40-fold between 2003 and 2011 versus 2.4-fold for the USA. The growth of Chinese meta-analyses was driven by genetics (110-fold increase in 2011 versus 2003). The HuGE Navigator identified 612 meta-analyses of genetic association studies published in 2012 from China versus only 109 from the USA. We compared in-depth 50 genetic association meta-analyses from China versus 50 from USA in 2012. Meta-analyses from China almost always used only literature-based data (92%), and focused on one or two genes (94%) and variants (78%) identified with candidate gene approaches (88%), while many USA meta-analyses used genome-wide approaches and raw data. Both groups usually concluded favorably for the presence of genetic associations (80% versus 74%), but nominal significance (*P*<0.05) typically sufficed in the China group. Meta-analyses from China typically neglected genome-wide data, and often included candidate gene studies published in Chinese-language journals. Overall, there is an impressive rise of meta-analyses from China, particularly on genetic associations. Since most claimed candidate gene associations are likely false-positives, there is an urgent global need to incorporate genome-wide data and state-of-the art statistical inferences to avoid a flood of false-positive genetic meta-analyses.

## Introduction

Meta-analyses are influential publications [1]. They can summarize evidence quantitatively across diverse disciplines and can inform decisions about the need for further research and/or practical implementations of the research findings [2]. The method’s popularity has surged in the last two decades with the advent of evidence-based medicine. Noticeably, the application of meta-analyses has extended globally, involving many other countries beyond the United States (US) and selected western countries. This trend reflects the globalization of scientific research, the deluge of published data in the current era, and the need for knowledge integration [3], [4]. Given the influence of meta-analyses in assessing the robustness of scientific evidence, there is a need to evaluate the volume of meta-analyses and their quality, specifically in the scientific fields which have undergone the most rapid expansion in production of data.

To obtain a better understanding of the expansion of the meta-analyses literature, in this paper we systematically mapped and evaluated the extent and pace of growth of the meta-analysis literature in biomedical science worldwide. The number of meta-analyses published annually approximately doubled in the last 5 years. We were intrigued to document a very rapid rise in the production of meta-analysis from China. Meta-analyses from the US (traditionally the key producer of meta-analyses in the past) less than doubled in the last 5 years. Conversely, meta-analyses from China outnumber those from the US in the current production, while very few meta-analyses came from China until a few years ago. The advent of Chinese meta-analysis production was driven primarily from genetics, a field that until recently was dominated by papers from the US and a few European countries. To understand the dynamics and patterns of this growing literature, we also performed a more in-depth evaluation of meta-analyses of genetic associations from China and the US to compare their methodologic characteristics.

## Methods

### Survey of Meta-analyses – Search Strategies

We searched PubMed (last search date September 1, 2012) for publications classified as type “meta-analysis” and performed also counts per publication year from 1995 until 2012. We generated separate counts for all meta-analyses worldwide, as well as those with listed affiliation from the People’s Republic of China (China [affiliation] NOT Taiwan [affiliation]) and, for comparison, those with affiliation from the US (USA [affiliation] OR US [affiliation] OR United States [affiliation]), since US has historically been the top producer of meta-analysis publications.

We then separated the meta-analysis publications according to field as follows. First, genetics-related meta-analyses were searched using the strategy “gene OR genetic OR polymorphism OR genome OR mutation OR haplotype”. Of those meta-analyses not captured with this strategy, we used the search “trial OR random* OR treatment” to identify treatment-related meta-analyses. Of those captured with neither of these two strategies, we used the search term “sensitivity” to identify meta-analyses of diagnostic performance. Of those not captured with any of these three searches, we used the search “cohort or case control” to identify other meta-analyses mentioning studies with such designs. All remaining meta-analyses were placed in a miscellaneous group.

Given that the greatest share of meta-analyses from China was identified in the genetics-related group, we also mapped the evolution of the number of meta-analyses in genetics (total, from China and from US for comparison). We also evaluated whether genetics-related meta-analyses from China are published in the English or Chinese language, as well as whether they address genetic associations of gene variants or other research gene-related questions.

The HuGE Navigator [5] (last update search performed January 13, 2013) was also used to map annually the evolution of the number of published meta-analyses on genetic associations of gene variants since 2000 around the world and the number of meta-analyses published per year was plotted for the 10 most prolific countries during 2000–2012.

### Comparative Evaluation of Recent Meta-analyses in Genetics

The field of genetics has experienced a paradigm transformation since 2005. Previously, most genetic studies followed a candidate gene approach: one or a few genes and variants thereof were chosen based on biological reasoning to test for association with some phenotype/disease of interest. More recently, genome-wide association studies (GWAS) have probed associations across the whole genome and claim discoveries only after rigorous replication and stringent criteria of multiplicity-adjusted significance are met [6], [7]. Large-scale agnostic studies have also permitted testing previously proposed candidate gene associations. In such evaluations the majority of associations proposed in the candidate gene era have not been replicated [8]–[11].

Given the large amount of genetic meta-analyses from the US and China, we compared genetic meta-analyses from the two countries to describe the current state of meta-analysis approach in the published literature. We evaluated 100 genetic association meta-analysis articles (50 from China and 50 from the US) published in 2012. We defined genetic association meta-analyses to include studies that use published and/or new data on candidate or GWAS-derived associations (newly proposed, or further validated) of genetic variants with any outcome/phenotype of interest. We included in this category, meta-analyses of variants with pharmacogenetic associations, and meta-analyses of Mendelian randomization provided they also addressed some clinical phenotype. We excluded meta-analyses of somatic mutations and of gene expression data. Articles selection was done by systematically screening through the meta-analyses published in 2012 in chronological order of PubMed indexing until we identified 50 eligible meta-analyses from each country group. For these 100 meta-analyses, we extracted the following information: journal of publication (so as to identify subsequently also the 2011 Journal Impact Factor from Thomson ISI); number of authors; language of publication; disease/phenotype (cancer, cardiovascular, infectious diseases, other disease, non-disease); type of data included (literature, investigators’ own, both); inclusion of any unpublished data other than those of the meta-analysis investigators; number of genes assessed (1,2,3, >3); number of genetic variants assessed (1,2,3, >3); any new associations proposed (yes/no – if no, whether the previously proposed genes that were probed had been derived from agnostic approaches (GWAS) (none [candidate genes only], some, all – the GWAS Catalog constructed by National Human Genome Research Institute (NHGRI) [12] was consulted to identify whether any of the gene-phenotype associations had been identified in GWAS); models used for data synthesis (fixed effect, random effects, both); relative risks or absolute differences reported in the abstract (relative risk, absolute difference, both, none); largest relative risk (more deviating from 1.00 in either direction) reported in the abstract; largest nominally statistically significant relative risk reported in the abstract; any nominally statistically significant results (*P*<0.05 or 95% confidence interval excluding the null) reported in the abstract (yes/no); any genome-wide statistically significant results (conventionally defined as *P*<5×10^−8^) reported in the abstract (yes/no); conclusion of the abstract regarding whether there is some association or not (yes/no); abstract suggesting differences in populations of different ethnicity/ancestry (yes/no); abstract suggesting significant associations only with a particular inheritance model, and if so, which; and any suggestions made in the abstract that more data are needed (yes/no). We also evaluated whether the meta-analyses included any data from GWAS (yes/no); whether the eligibility criteria aimed to include data from Chinese-language studies (yes/no); whether literature searches included Chinese biomedical literature databases [13] or were limited to western databases such as PubMed and EMBASE; and whether any Chinese-language studies were indeed included in the meta-analysis calculations, and, if so, how many. For comparison, we also assessed whether studies in any other language besides English/Chinese were considered eligible.

We compared the two groups using chi-square test with Yates’ correction, Fisher’s exact test, Freeman-Halton or chi-square test adjusting for trend, and Mann-Whitney U test, as appropriate. With n = 50 meta-analyses in each group, and assuming that 20% of the US meta-analyses would assess candidate gene variants, we had at least 90% power to find a significant difference at alpha = 0.05, if the proportion of meta-analyses from China addressing candidate genes were 50% or higher [14]. One comparison used all 100 meta-analyses; a further analysis focused only on meta-analyses that addressed only genetic variants that have not been validated in GWAS, since the large majority of these associations are likely to be spurious [8], [9], [10], [15], [16]. All P-values are two-tailed.

## Results

### Number of Meta-analyses

As of September 1, 2012, PubMed tagged a total of 34,238 publications as meta-analyses. The number published each year since 1995 is shown in Table 1. Overall, there is a substantial growth over time, with 11-fold increase in the annual number between 1995 and 2011. In the same time frame, the annual volume of items indexed in PubMed has approximately doubled (n = 443,543 in 1995, n = 865,176 in 2011).

**Table 1:** Meta-analyses in PubMed According to Publication Year.

Year	All	China	US
1995	429	0	165
1996	482	1	197
1997	596	3	250
1998	639	0	235
1999	741	0	305
2000	849	2	335
2001	948	3	366
2002	1078	11	400
2003	1289	19	401
2004	1594	28	467
2005	2063	33	541
2006	2331	77	681
2007	2594	97	696
2008	2773	179	756
2009	3229	302	774
2010	3904	540	896
2011	4739	828	965
2012 (until search)	2270	464	446

US: United States. When a paper in published as Epub and then final publication, the year of the final publication is counted. The same applies to data in tables 2 and 3.

The United States was the most common country affiliation accounting for 8,886 of the 34,238 meta-analyses (26%). There is a clear decline in the proportion of the total represented over time (from 38% in 1995 down to 20% in 2012). Conversely, China has emerged as a dominant publisher of meta-analyses. When all years are considered, it lagged behind the US with a total of 2,587 (8%) publications overall; nevertheless, the rate of growth in meta-analyses is rapid. Prior to 2003, China contributed less than 1% of the total meta-analyses. Between 2003 and 2011, the annual number of Chinese meta-analyses increased over 4000%, versus 140% for the US. In 2012 meta-analyses from China surpassed meta-analyses from the US (21% versus 20% of the total).

The sheer proportional increase in the number of published meta-analyses is markedly specific for China. Japan accounts for 1.5% of meta-analyses published (1.3% when limited to 2012 alone), and no other Asian country accounts for more than 1% of meta-analyses published over all time or focusing on 2012 only. Several European countries have long published meta-analyses and continue to do so with modest increases in the number of meta-analyses published per year, but currently their relative contribution is far less prominent than US or China (not shown in detail).

### Types of Meta-analyses

As shown in Table 2, the majority of meta-analyses overall pertain to clinical trials and treatment topics (68%). Genetics-related meta-analyses accounted for 11% of the total when all years are considered and substantially higher proportions in later years (19% in 2012).

**Table 2:** Different Types of Meta-analyses Overall and from China.

	Any publication year		Published in 2012(until September 1 search)	
Search strategy	All	China (%)	All	China (%)
Gene OR genetic OR polymorphism OR genome OR mutationOR haplotype	3631	942 (26)	441	210 (48)
Trial OR randomi* OR treatment	23529	1307 (6)	1388	195 (14)
Sensitivity	1298	125 (10)	99	24 (24)
Cohort OR case control	1151	86 (7)	62	10 (16)
Miscellaneous meta-analyses	4629	127 (3)	280	25 (9)
ALL META-ANALYSES	34238	2587 (8)	2270	464 (21)

Among genetics-related meta-analyses, production from China accounts for 26% of all meta-analyses when all years are considered and approximately half of the published papers (48%) in 2012. In 2012, among papers from China, genetics-related meta-analyses outnumber in absolute numbers meta-analyses of clinical trials and treatments; conversely meta-analyses of clinical trials and treatments remain more than 5-times more common than genetics-related meta-analyses among papers coming from countries other than China (n = 1191 versus n = 231).

### Growth of Genetics-related Meta-analyses

Table 3 shows the yearly publication of genetics-related meta-analysis from 1995 to 2012 for all countries, China, and the US. Compared to 1995, there was a 64-fold increase in the number of genetics-related meta-analyses in 2011. The contribution from the US was particularly dominant from 1995 to 1997, where it contributed approximately half of the total papers published. The proportion of US contribution has gradually declined to 14%. The converse is observed for China where it contributed no genetics-related meta-analysis from 1995 to 2002. From 2003, a rapid growth occurred with what appeared to be a doubling in the annual number of contributions per year. More specifically between 2003 and 2011, there was a 110-fold increase in publications from China. In 2012 (based on papers indexed in PubMed through August), genetics-related meta-analyses from China were 3.5-fold more prevalent than meta-analyses from the US (n = 210 versus n = 61).

**Table 3:** Genetics-related Meta-analyses.

Year	All	China	US
1995	12	0	6
1996	9	0	5
1997	31	1	15
1998	24	0	4
1999	40	0	13
2000	50	0	18
2001	61	0	23
2002	56	0	17
2003	91	3	22
2004	130	5	36
2005	155	9	37
2006	213	21	51
2007	245	26	56
2008	286	40	66
2009	394	81	75
2010	590	221	105
2011	774	327	137
2012 (until search)	441	210	61

US: United States.

With very few exceptions, the genetics-related meta-analyses from China indexed in PubMed are published in English-language journals (902/942, 96%). Following a suggestion raised during peer-review, we assessed whether any of the meta-analyses from China had been published in duplicate, in both the Chinese and English language. Careful scrutiny of a sample of 15 Chinese-language meta-analyses on genetic associations showed that for 2 of them, a corresponding meta-analysis on the same variant and phenotype and with partially overlapping authors had been published also in an English-language journal within <1 year time-difference. In one pair, the Chinese-language meta-analysis [17] concluded that GSTM1 is significantly associated with colorectal cancer risk, while the English-language meta-analysis [18] concluded that GSTM1 is not significantly associated with colorectal cancer risk, while other metabolic enzyme genetic polymorphisms were associated. In the other pair, the Chinese-language meta-analysis [19] found associations for SLC11A1 (formerly NRAMP1) gene polymorphisms and tuberculosis susceptibility focusing on East Asian populations, while the English-language meta-analysis [20] included diverse ethnic groups and also found significant associations for the same variants in East Asian populations but not with identical effect sizes.

**Figure 1:**
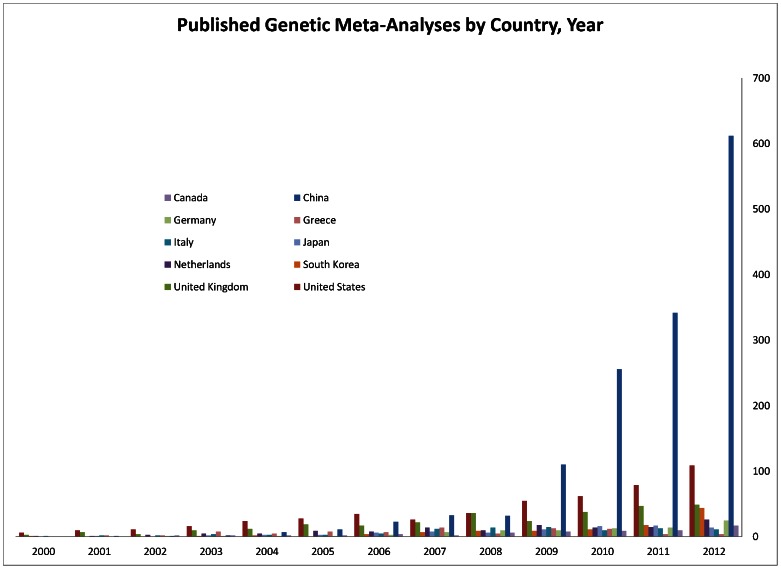
Annual number of meta-analyses of genetic associations for the 10 most-prolific countries in the period 2000–2012; data are derived from HuGE Navigator (last update January 13, 2012).

The large majority of the Chinese genetics-related meta-analyses addressed genetic associations of gene variants (95/100 among the latest 100 meta-analyses indexed in PubMed, 90/95 evaluating only a single gene). Figure 1 shows the data from HuGE Navigator on the ten most prolific countries of genetic association meta-analyses with data updated to the end of 2012. Consistent with the PubMed data, the genetic association meta-analyses from China followed a geometric growth, while the US slowed, and other countries contributed comparatively few meta-analyses. Among Asian countries, South Korea is fourth in number of genetic association meta-analyses in 2012, but very far from both China. By the end of 2012, there were 612 genetic association meta-analyses published from China in that single year, versus only 109 from the US, 49 from the United Kingdom, and 44 from South Korea. No other country produced more than 30 genetic association meta-analyses in 2012.

### Comparative Evaluation of Genetic Association Meta-analyses from China and US

Table 4 shows the distribution of articles published in 2012 for China and US by selected characteristics. As shown, meta-analyses from China and the US differed significantly in several features, including source of data, number of genes studied, and type of approaches (candidate-gene or GWAS). Ninety-two percent of Chinese articles performed their meta-analyses using only data abstracted from the literature as compared to 28% from the US. Conversely, seventy-two percent of US investigators included their own genetics data compared to 8% of Chinese investigators. Unpublished data beyond those of the investigators participating in the meta-analysis were rarely included in any meta-analysis (n = 1 in China-based articles, n = 0 in US-based articles). Meta-analyses from the US tended to examine more genes than ones from China. A majority (88%) of Chinese meta-analyses addressed gene variants that were previously identified using a candidate-gene approach. In comparison, US meta-analyses were evenly split between candidate-genes and GWAS-derived genes and/or new GWAS discoveries.

**Table 4:** Comparison of Characteristics of Genetic Association Meta-analyses Published in 2012 from China and US.

Characteristic		China	US	*P* value
Journal impact factor, median (IQR)		2.541	6.575	<0.001
Number of authors	1	2	0	<0.001
	2	2	2	
	3–5	18	8	
	6–10	25	5	
	11–50	3	21	
	>50	0	14	
English language		49	50	1.00
Disease/Phenotype	Cancer	23	8	0.001
	Cardiovascular	6	7	
	Infectious diseases	2	0	
	Other disease	14	17	
	Non-disease	5	18	
Type of data included	Literature	46	14	<0.001
	Investigators’ own	0	12	
	Both	4	24	
Other unpublished data		1	0	1.00
Number of genes assessed	1	44*	13	<0.001
	2	3	1	
	3	0	0	
	>3	3	36	
Number of genetic variants assessed	1	27	10	<0.001
	2	12	1	
	3	3	1	
	>3	8	38	
New genes/variants proposed	Yes	2	18	<0.001
	No	48	32	
If no, proposed genes from GWAS	None	45	16	<0.001
	Some	0	0	
	All	5	16	
Metrics reported in the abstract	Relative risk	43	15	<0.001
	Absolute difference	0	1	
	Both	0	0	
	None	7	34	

US: United States.

* Includes a study that evaluated a single intergenic variant.

As shown in Table 5, there was a significant difference in China- versus US-based meta-analyses in the use of fixed and/or random effects models for data synthesis (*P*<0.0001), because US articles were mostly GWAS where fixed effects analyses are long established as the standard method applied for making discoveries. The largest relative risks and the largest statistically significant relative risks were not significantly different in the two groups of meta-analyses, when all 100 meta-analyses were considered. The largest significant risks were larger in China- rather than US-based meta-analyses, when limited to the set that did not include GWAS-validated genes (median 1.81 v 1.21, *P* = 0.036). Both groups of meta-analyses claimed significant results in the abstract (76% versus 82% in China versus US), but genome-wide significant findings were observed predominantly in US meta-analyses (n = 23), with only one exception in the China group (*P*<0.001 for the China-US comparison). Both groups usually concluded favorably for the presence of genetic associations (80% versus 72%). There was a suggestion that the Chinese group was more likely to invoke ethnicity/ancestry differences (24% versus 8%, *P* = 0.054) and possibly also associations that are specific to an inheritance model (8% versus 2%) in their discussion of the results, but these differences were not nominally statistically significant.

**Table 5:** Results and Conclusions of Genetic Association Meta-analyses Published in 2012 from China and US.

		All meta-analyses		Not GWAS genes*	
		China	US	China	US
		N = 50	N = 50	N = 45	N = 16
Statistical model for synthesis**	Fixed effect only	1	32	0	5
	Random effects only	3	7	2	5
	Both fixed and random	46	11	43	6
	*P* value	<0.001		<0.001	
Largest relative risk in abstract	Median, IQR	1.75, 1.25	1.49, 1.74	1.75, 1.26	1.21, 2.08
	*P* value	0.86		0.078	
Largest significant relative risk in abstract	Median, IQR	1.81, 1.31	1.66, 1.76	1.81, 1.31	1.21, 2.08
	*P* value	0.68		0.036	
Significant results in the abstract		38	41	33	9
	*P* value	0.62		0.22	
Any GWS results in the abstract		1	23	0	0
	*P* value	<0.001		1.00	
Abstract conclusions on associations	Presence of association	40	37***	35	8
	*P* value	0.63		0.055	
	Ethnicity/ancestry differences	12	4	11	3
	*P* value	0.054		0.74	
	Inheritance model-specific	4	1	4	1
	*P* value	0.36		1.00	
	More evidence is needed	15	9	13	6
	*P* value	0.24		0.54	

IQR: interquartile range, GWS: genome-wide significant, US: United States.

* Includes only data from meta-analyses that include only candidate genetic variants that have not been validated in GWAS **Methods combining p-values or z-scores, or pooled analysis, were counted as equivalent to fixed effects; Mixed effects model was counted as random effects *** some studies with significant reported results are counted here, even if no concluding statement was made.

### Inclusion of Data from GWA Studies and from Chinese-language Literature

One study from China included data from GWA studies, while GWAS data was included in 31 of the 50 meta-analyses from the US (*P*<0.001). Among the 48 studies from China and 12 from the US that performed literature searches, the eligibility criteria aimed to include data from published Chinese-language studies in 38 meta-analyses versus 8 respectively (*P* = 0.448). Literature searches perused Chinese-language biomedical literature database in 21 versus 1 meta-analyses, respectively (*P* = 0.041). Chinese-language studies were included in the meta-analysis calculations in 20 versus 2 meta-analyses (*P*<0.001 for all studies, *P* = 0.180 for studies using literature searches). A total of 65 Chinese-language genetic association studies were included in the calculations of 20 China-based meta-analyses, and 9 meta-analyses included > = 3 Chinese-language studies. In contrast, a total of 9 Chinese language genetic association studies were included in the calculations of 2 US-based meta-analyses. Articles in languages other than English and Chinese were considered eligible according to the Methods section in 29 meta-analyses from China and 7 meta-analyses from the US among the 48 and 12 meta-analyses respectively that performed any literature searches (*P* = 1.00).

## Discussion

Our empirical overview of the meta-analyses literature shows a rapid increase in meta-analysis studies worldwide. The increase is most prominently seen in China, specifically in genetics. Chinese publication of genetic association meta-analyses was more than 5 times higher than US-published meta-analyses during 2012 and currently China dominates the global production of papers in this field. In the last 8 years, the annual production of meta-analyses of China has increased 40-fold overall with a 110-fold increase in genetics.

The vast majority of the genetics meta-analyses from China resulted from combining data from association studies evaluating one or two specific gene variants that had been proposed in the candidate gene era. These studies based significant results on nominal significance (*P*<0.05) rather than genome-wide significance thresholds. US investigators tended to include their own genetic data in the meta-analyses whereas Chinese papers typically did not address GWAS-proposed variants. Many of the China-based meta-analyses also include published Chinese-language studies that are not indexed in western databases.

China is becoming an increasingly important player in biomedical research as illustrated in the exponential contribution of published works from Chinese investigators. To our knowledge, the meta-analysis literature from China has not been comparatively evaluated previously in a systematic fashion. One exception is an empirical evaluation of systematic reviews of acupuncture from China. The evaluation suggested that the systematic reviews conducted lacked rigor in appraising the risk of bias in included studies [21]. Likewise, empirical investigations in some other fields, including single genetic association studies of candidate genes, clinical trials, and randomized trials on acupuncture have suggested that Chinese studies present a prominent excess of significant results [22]–[25] that requires cautious interpretation.

Strong evidence has accumulated on the low replication rates of past candidate gene associations, when these associations are evaluated in large-scale consortia with agnostic testing of gene variants across the whole genome [8], [9], [15]. Thus, one may infer that the large majority of significant associations proposed in meta-analyses of candidate gene studies are likely to perpetuate false-positive findings. Therefore, it is likely that the majority of China-produced genetic meta-analyses are reaching false-positive conclusions. The reasons for this may be manifold, but we have identified some potential explanations.

First, China-based meta-analyses do not employ data from GWA studies. This may be due to the fact that these data are not readily available to a wider public, or they may need approval processes which hinder access from Chinese meta-analysts. In some occasions, the genetic variants of interest may not be captured by agnostic platforms, although this is not common with current GWA platforms that have very high genome coverage and imputation should make this concern even less important [26].

Second, China-based meta-analyses tended to focus on testing single genes and gene variants that were proposed in the candidate-gene era. This approach does not address genetic variants that have emerged with far stronger statistical support from large-scale consortia performing GWAS [27]. Overall, these meta-analyses have not followed the evolution of human genome epidemiology in the direction of GWA studies, e.g. currently large-scale synopses of genetic association studies routinely try to incorporate the GWA data, which are typically the largest datasets in each field [28], [29]. Lack of inclusion of GWA data in the current meta-analysis literature may not be necessarily exclusive to China. Meta-analysts from other countries may also continue to perform candidate-gene meta-analyses. Unfortunately, the number of such meta-analyses from other countries is extremely small compared to China to allow a meaningful evaluation.

Third, many China-based meta-analyses performed more exhaustive literature searches than US-based meta-analyses and included data from Chinese-language publications that are often indexed only in Chinese literature databases [13]. While inclusiveness is commendable in principle, previous experience suggests that language bias could affect the results in different ways depending on the field involved [4], [30], [31]. In some disciplines, it may be preferable to exclude rather than to include data from specific countries. It was demonstrated [25] that there is a prolific Chinese language literature on genetic association studies addressing variants of the candidate gene era and this literature might be biased towards reporting of statistically significant results [25]. This was not due to the quality of the studies based on the reported features of their methods and conduct [25], but may be due to selective reporting of “positive” results. Under such circumstances, inclusion of these data may propagate further false-positive results at the meta-analysis level.

We should acknowledge some limitations in our study. We did not assess in depth the quality of the evaluated meta-analyses, which could further explain the differences between China and US meta-analyses. Quality of published genetic association meta-analyses is difficult to assess as there is no guarantee that reported features accurately reflect real practices adopted during the conduct of the meta-analysis [32]. As previously observed [25], reported quality of Chinese genetic association studies was comparable to studies performed in other countries. Moreover, quality comparisons between China and the US might not be meaningful or even feasible because the types of research endeavors differ between the two countries with minimal overlap, e.g. meta-analyses of published data on candidate gene associations versus consortium analyses of GWA data. Eventually, one needs to assess the essential features and reliability of a meta-analysis regardless of its country of origin. However, the extremely rapid increase in Chinese meta-analyses of candidate gene variants is a phenomenon that cannot be underestimated and it has no parallel in any other country to-date.

Allowing for these caveats, our empirical evaluation documents an extraordinary, geometric growth of the meta-analysis literature produced by authors from China in the last few years, with strong emphasis on genetic associations. This surge in meta-analysis applications presents conflicting issues. On the one hand, meta-analyses of data abstracted from the literature and from candidate-gene studies may propagate an epidemic of false claims for candidate gene associations. Conversely, the extraordinary scientific potential of China can offer tremendous impetus to evidence-based medicine in general and genetics more specifically, if it is appropriately harnessed. Efforts should be made to familiarize Chinese meta-analysts with the newer waves of genetic studies and improved access to large-scale consortium databases and active participation in such consortia [33] may be useful steps in this direction. China is already a leading power in modern genomic technologies, with unparalleled sequencing capacity and is already assuming a leading role in the emerging omics fields [34]–[36]. Meta-analyses could also be facilitated to reach higher levels of reliability.
